# An optimized force-triggered density gradient sedimentation method for isolation of pelage follicle dermal papilla cells from neonatal mouse skin

**DOI:** 10.1186/s13287-023-03343-2

**Published:** 2023-05-24

**Authors:** Lijuan Du, Yuyang Gan, Bowen Zheng, Junfei Huang, Zhiqi Hu, Yong Miao

**Affiliations:** Department of Plastic and Aesthetic Surgery, Nan Fang Hospital of Southern Medical University, 1838 North Guangzhou AV, Guangzhou, Guangdong China

**Keywords:** Pelage follicle, Dermal papilla, Force-triggered density gradient sedimentation, FDGS

## Abstract

**Background:**

The dermal papilla cells are a specialized population of mesenchymal cells located at the base of the hair follicle (HF), which possess the capacity to regulate HF morphogenesis and regeneration. However, lack of cell-type specific surface markers restricts the isolation of DP cells and application for tissue engineering purposes.

**Methods:**

We describe a novel force-triggered density gradient sedimentation (FDGS) method to efficiently obtain purified follicular DP-spheres cells from neonatal mouse back skin, utilizing only centrifugation and optimized density gradients.

**Results:**

Expression of characteristic DP cell markers, alkaline phosphatase, β-catenin, versican, and neural cell adhesion molecules, were confirmed by immunofluorescence. Further, the patch assays demonstrated that DP cells maintained their hair regenerative capacity in vivo. Compared with current methods, including microdissection and fluorescence-activated cell sorting, the FDGS technique is simpler and more efficient for isolating DP cells from neonatal mouse skin.

**Conclusions:**

The FDGS method will improve the research potential of neonatal mouse pelage-derived DP cells for tissue engineering purposes.

**Supplementary Information:**

The online version contains supplementary material available at 10.1186/s13287-023-03343-2.

## Introduction

The dermal component of hair follicles (HFs) consists of the dermal papilla (DP) and sheath. DP contained a specialized population of mesenchymal cells located at the base of the HF, DP cells, which possess the capacity to regulate HF morphogenesis and regeneration [1–3]. In addition, the DP cells can re-populate the dermal cell population to maintain its integrity [4]. Due to this regenerative capacity, DP cells are a promising source for autologous cell therapies in the field of tissue engineering [5].

For research purposes, murine vibrissae are a common source of DP cells due to their relatively large size [6–8]. However, on average, an adult mouse has only 15–20 vibrissae hair follicles. The hair growth cycles of murine vibrissae can vary, including a rapid anagen and a short telogen phase [9, 10]. Considering their limited quantity and variable growth cycles, the current model for investigating DP biology need to be improved. Alternatively, pelage HFs are more abundant with a more stable growth cycle. Previous report showed that in adult mouse, the expression of sox2 in pelage DPCs was mostly negative, while positive in vibrissae DPCs. However, in neonatal mouse, both vibrissae and pelage DPCs expressed sox2, and there was no other markers that can be distinguished. At present, it is believed that there are structural differences, such as blood sinuses(showed in vibrissase). Pelage HFs are an alternative source of DPs for investigating developmental biology and regenerative medicine [11–13].

To date, there are two main isolation methods for HFs: microdissection and enzyme digestion. Due to the small size of pelage HFs, microdissection cannot be used to obtain DP cells from murine skin [14]. The enzyme digestion method can be combined with fluorescence-activated cell sorting (FACS) to isolate DP cells [15]. The molecular profile of DP cells includes markers, such as alkaline phosphatase (ALP), β-catenin, versican, and neural cell adhesion molecules (NCAM), as well as pluripotent markers Sox2, c-Myc and Klf4. This evidences suggest that DPs have a remarkable level of subpopulation heterogeneity [16–19]. However, DP cells isolated by a single marker may only represent a certain subpopulation. Most classical experiments need to be using multiple transgenic reporters mice (such as Sox2, LEF1 and ITGA9 et al.) according to the results of previous single cell sequencing, which requires high technology and funds.

Herein, we demonstrate a simple method named force-triggered density gradient sedimentation (FDGS), which only based on the biological characteristics (such as cellularity and density) that allows the separation of cells depending on their density. Intact naïve follicle components (NFC) and DP spheres can be isolated and purified by removing unwanted single cells using Ficoll-mediated density gradient centrifugation. In our previous study, we also successfully isolated pelage DP cells from adult mice by using a modified FDGS method [20]. The FDGS method compensates for difficulty of HF-DP cell isolation, improving their research potential for tissue engineering purposes.

## Materials and methods

### Animals

Neonatal pups (postnatal day 2, P2) and adult (4-week-old) C57BL/6 J mice and adult (4 week old) athymic nude mice (BALB/cAJcl-nu) were purchased from the Experimental Animal Center of Southern Medical University (Guangzhou, China). The animal study protocol was approved by the Experimental Ethics Center of Southern Medical University and performed according to the “Guidelines for the Care and Use of Laboratory Animals”. Mice were placed in the SPF facility of the animal center of Experimental Animal Center of Southern Medical University and reared at room temperature 25 ± 2 °C, relative humidity 65 ± 2%, and a 12 h light / dark cycle, and were fed with regular food and water. Ten neonatal C57BL/6 J mice were used in each DP-isolation experiment. Six athymic nude mice were used in each HF-reconstitution experiment. Adult (4-week-old) C57BL/6 J mice were used in histological analysis. Each experiment was repeated at minimum three times.

### Preparation of dermis

Euthanize mouse pups using a CO_2_ chamber for at least 20 min. Skin specimens were harvested from P2 pups. The skin was incubated with 0.1% dispase (Sigma, St Louis, MO, USA) at 4 °C overnight, and then subsequently incubated with 0.05% trypsin–EDTA (Gibco, Carlsbad, CA, USA) for 1 h at 37 °C. The epidermis and dermis were gently separated by scraping with the blunt side of a surgical knife under sterile conditions. The dermis was cut into small pieces with ophthalmic scissors and minced by a razor blade until the dissociated dermal tissue could be pipetted using a Pasteur pipette. The dermal cells were isolated by incubation with pre-warmed 0.2% collagenase I (Sigma) for 2 h at 37 °C, followed by gentle mixing for 10 min. Fetal bovine serum (FBS; Gibco) was added to the enzymatic solution at a 10% (v/v) final volume, and then gently pipetted for 5 min to dissociate the cell aggregates.

### Isolation and purification of the naïve follicle component (NFC)

Dissociated cells were centrifuged at 150 × g for 3 min to separate single dermal fibroblasts (DFs), which were then removed by discarding the supernate. Unless otherwise stated in this protocol, all centrifugation supernates were discarded, and the pellets were retained. The pellet was washed twice with 10% FBS/DMEM by resuspension and centrifugation at 30 × g for 3 min.

**Fig. 1:**
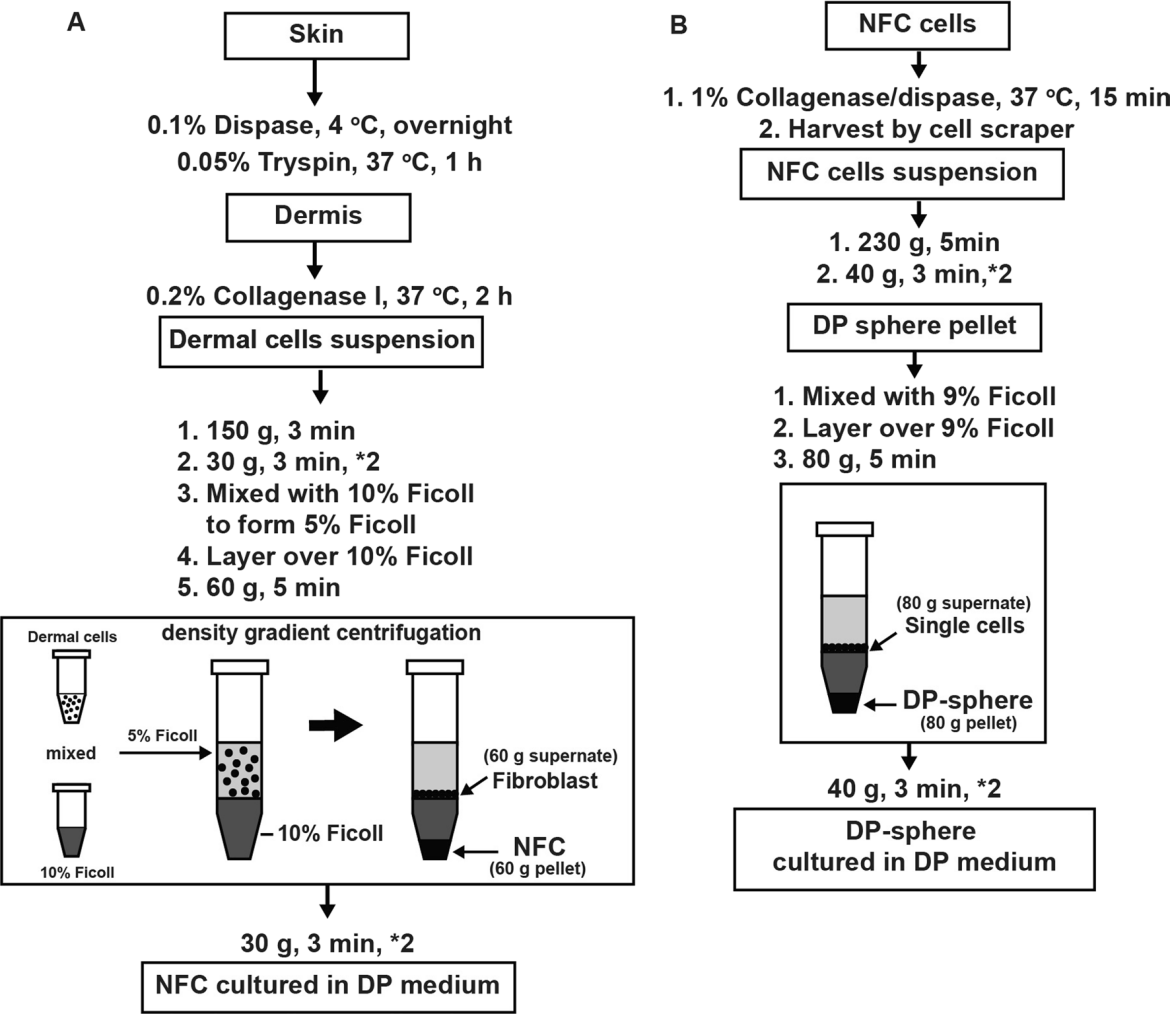
Schematic representation of the FDGS isolation method. **A** NFC isolation procedure from dermal tissues of P2 C57BL/6 J mice. **B** DP cell isolation procedure from day 2 cultured NFC cells

For NFC separation, the pellet was resuspended in 10% FBS/DMEM (4 mL/5 pups) and mixed with equal volumes of 10% (v/v) Ficoll PM400 (Sigma) to from 5% Ficoll mixed cell suspension. The 5% Ficoll mixed cell suspension was layered over 5 mL 10% Ficoll and centrifuged at 60 × g for 5 min. The pellet was washed twice in 10% FBS/DMEM and then centrifuged at 30 × g for 3 min to remove the remaining Ficoll. NFCs were resuspended in Follicle Dermal Papilla Cell Growth Medium (DP medium; Creative Bioarray, Shirley, NY, USA), containing basic fibroblast growth factor (0.5 ng/mL), insulin (2.5 μg/mL), and bovine pituitary extract (BPE; 104 μg/mL), supplemented with FBS (4% v/v), and then were plated onto 10 cm^2^ cell-culture dishes. Under these culture conditions, contaminating keratinocytes were non-proliferative as confirmed by in vitro observation. NFCs were monitored using an Olympus FluoView FV10i confocal laser scanning microscope (Olympus, Tokyo, Japan). Z-stacks were acquired in 1 μm planes at 1024 × 1024 dpi resolution. Time-lapse images were continuously acquired at 10 min intervals. The method is summarized in Fig. 1A.

### Isolation and purification of DP-spheres

Two days after culture, DP medium was discarded and the adherent NFC cells were washed twice with phosphate-buffered saline (Gibco). A pre-warmed 1% collagenase/dispase solution (Roche, Basel, Switzerland) was added and then incubated for 15 min at 37 °C. The cells were harvested using a cell scraper. NFCs were centrifuged at 230 × g for 5 min to remove remaining enzyme solution. The pellet was washed twice by resuspension in 10% FBS/DMEM and centrifugation at 40 × g for 3 min to remove single cells. To purify DP-spheres, the pellet was resuspended in 10% FBS/DMEM and mixed with equal volumes of 10% Ficoll. The mixed cell suspension/5% Ficoll was layered over equal volumes of 10% Ficoll and centrifuged at 80 × g for 5 min. The DP pellet was washed twice by resuspension in 10% FBS/DMEM and centrifugation at 40 × g for 3 min. The isolated DP-spheres were resuspended in DP medium and plated onto T25 flasks. The culture medium was changed every 3 days. When the DP explants began to merge, the cells were passaged in 0.25% trypsin/EDTA (Gibco). The method is summarized in Fig. 1B.

### Statistical analysis

All of the experimental data were expressed as means ± standard deviation from three independent experiments. A two-way analysis of variance (ANOVA) analysis was performed to determine significant differences between sample groups using the SPSS17.0 software. A *p*-value < 0.05 was considered as statistically significant. All graphs were plotted using GraphPad Prism 6 (GraphPad Software, Inc., La Jolla, CA, USA).

## Results

### Characterization of neonatal pelage HF

**Fig. 2:**
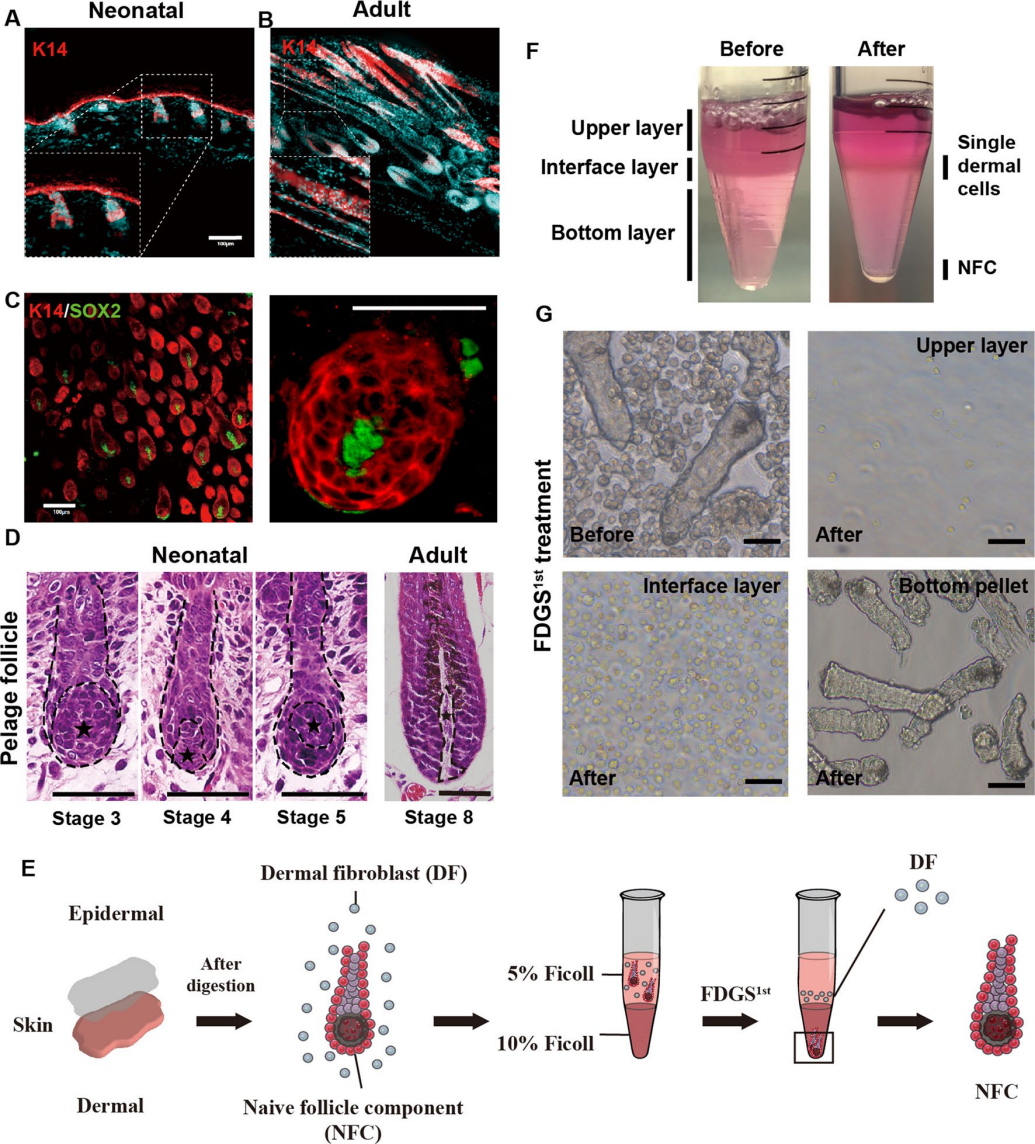
Characterisation of NFCs in vivo and after initial FDGS treatment. Two-day-old neonatal mouse (**A**) and two-week-old Adult mouse (**B**) dorsal skin immunolabelled with K14 antibody (red). Inserts show higher magnification views of the epidermal component. **C** Confocal micrographs of the neonatal mouse skin whole-mount immunolabelled with K14 (red) and Sox2 (green). Inserts show higher magnification views of DP. All sections with DAPI nuclear counterstain (blue). Scale bars: 100 μm. **D** H&E stain of dorsal skin showing HF morphogenesis stage and DP sphere formation. Stage 3: Dermal fibroblasts form a rounded DP cell. Stage 4: DP cells are greater in length than width and are enclosed by > 50%. Stage 5: DP cells are almost completely encapsulated and ball-shaped. Asterisks denote DP-spheres. **E** The schematic diagram of FDGS 1st procedure. **F** Photographs of a bilayer discontinuous Ficoll density gradient overlayed with dermal mixed cells in medium before and after centrifugation. **G** Representative images of NFC, before and after FDGS1st treatment, show a significant reduction in non-NFC single cells. Scale bars: 50 μm

To determine whether the FDGS isolation method is suitable for neonatal pelage HF, we detected the HF epithelial component in neonatal backskin. The outer root sheath cells were labeled with keratin 14 (K14). In P2 neonatal mice, the hair germ of dorsal HFs was immature with the most proximal part located in the dermis (Fig. 2A). Compared with neonatal HFs, dorsal HFs in 4-week-old adult mice were fully mature and at maximal length (Fig. 2B). K14 positive cells extending into the subcutaneous muscle layer, completely enclosing the DPs, and with the hair shaft emerging through the epidermis. As previous reported, Sox2 was expressed in almost all DPs (Fig. 2C). Sox2 co-staining with K14 further demonstrated the relationship between epithelial component and DP sphere position. Additionally, a haematoxylin and eosin (H&E) stain also confirmed that the neonatal HFs were undergoing morphogenesis. Most of the HFs were at stage 3–5, and the bulb was not yet enclosing the DPs (Fig. 2D). The lesser epithelial component involvement and greater undifferentiated cell subpopulation in the immature hair follicle suggested that neonatal mouse skin pelage HFs were more suitable for DP sphere isolation.

### Characterisation of isolated NFCs

The FDGS isolation method enabled efficient digestion of dermal collagen and release of non-DP cell types (including fibroblasts, vascular endothelial cells) [21]. Promisingly, the integrity of pelage NFC micro-tissue could be retained by this technique. Inspired by reports of tumor cells, mesenchymal stem cell and pancreatic islet purification [22–24], we sought to introduce Ficoll Density gradient mechanism and optimize the Ficoll concentration for optimal NFC separation. The first Ficoll density gradient sedimentation (FDGS 1^st^) isolation of NFC is shown schematically in Fig. 2E. After FDGS^1st^, the purified pelage NFC micro-tissue pelleted at the bottom of the tube, while fibroblasts settled in interface layers (Fig. 2F, G). Additional file 1: Fig. S1 summarizes the effects of different Ficoll concentrations on NFC separation. The most efficient isolation of NFC micro-tissue was achieved with a 10% concentration of Ficoll and decreased in a dose-dependent manner, corresponding to 15% and 20% Ficoll.

**Fig. 3:**
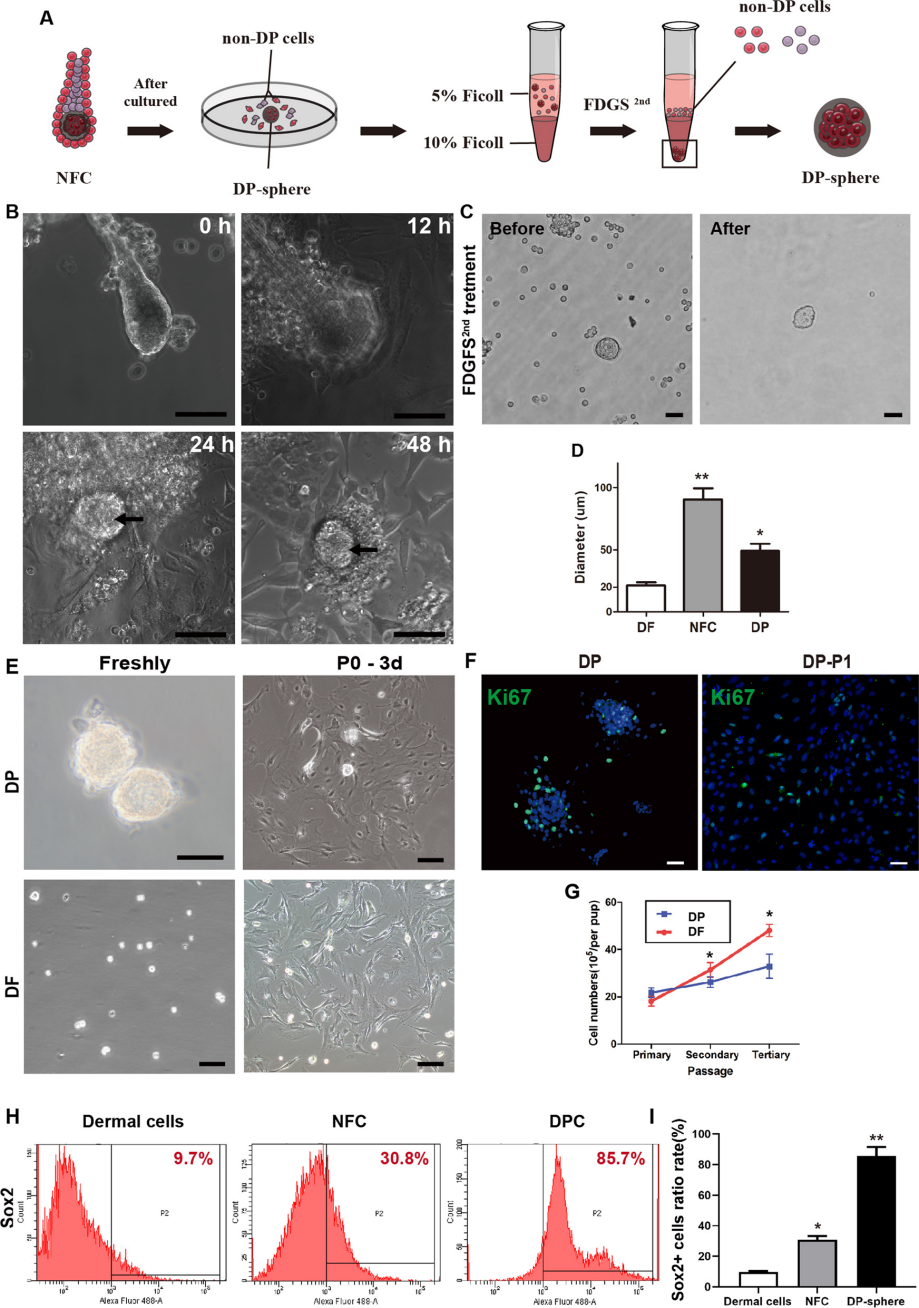
Characterisation of DP-spheres after second FDGS treatment. **A** The schematic diagram of FDGS 2st procedure. **B** Snapshots of live-imaging of NFC cultures after 48 h. Loss of three-dimensional follicle structure after 48 h of NFC culture. Single optical sections showing a single NFC at four consecutive time points (0, 12, 24, and 48 h). Arrows indicate DP-spheres. **C** Freshly isolated DP-spheres, before (left) and after (right) FDGS2nd treatment. **D** The transverse-section diameter of obtained DFs, NFCs, and DP-spheres. **E** Phase-contract microscopic images of freshly isolated DP-spheres and after 3 days of culture, respectively. DF cells were used as the control. **F** Isolated DP-spheres contained Ki67-positive (green) dividing cells after 2 days of culture. Scale bars: 50 μm. **G** Quantification of DP and DF cell proliferation rates after three passages in culture. **H** FACS analysis of Sox2^+^ cells in dermal cells, NFC and DP cells by flow cytometry.(G) Quantification of the percentage of Sox2^+^ cells in dermal, NFC and DP cells. (*p < 0.05)

After isolation and purification, the NFCs were plated onto culture dishes in DP medium (Fig. 3B). In general, > 80% of the NFCs attached within 24 h of culture and appeared to lose their initial three-dimensional (3D) follicular structure. These cells displayed a flat, polygonal morphology and formed various NFC cell emplanes. Forty-eight hours after culture, the almost NFC explants enlarged and lost their 3D structure. However, some clusters still had round-shapes and clear boundary with the surrounding area. We hypothesized that the round-shapes clusters was DP-spheres which due to the presence of basement membranes limited cell migration.

### *Characteristics of isolated DP-spheres *in vitro

Considering the anchorage-dependent and migration ability of DP-spheres, we predicted that NFCs would be more suitable for isolation after 48 h of culture. The second Ficoll density gradient sedimentation (FDGS^2nd^) isolation of DP-sphere is shown schematically in Fig. 3A. After FDGS^2nd^, the purified DP-spheres maintained their original spherical structure and presence of high cell density clump (Fig. 3C). The transverse-section diameter of the NFCs was 90.43 ± 9.04 μm, compared with 49.29 ± 5.59 μm for DP-spheres and 21.43 ± 2.44 μm for dermal fibroblasts (DF) (Fig. 3D).

**Fig. 4:**
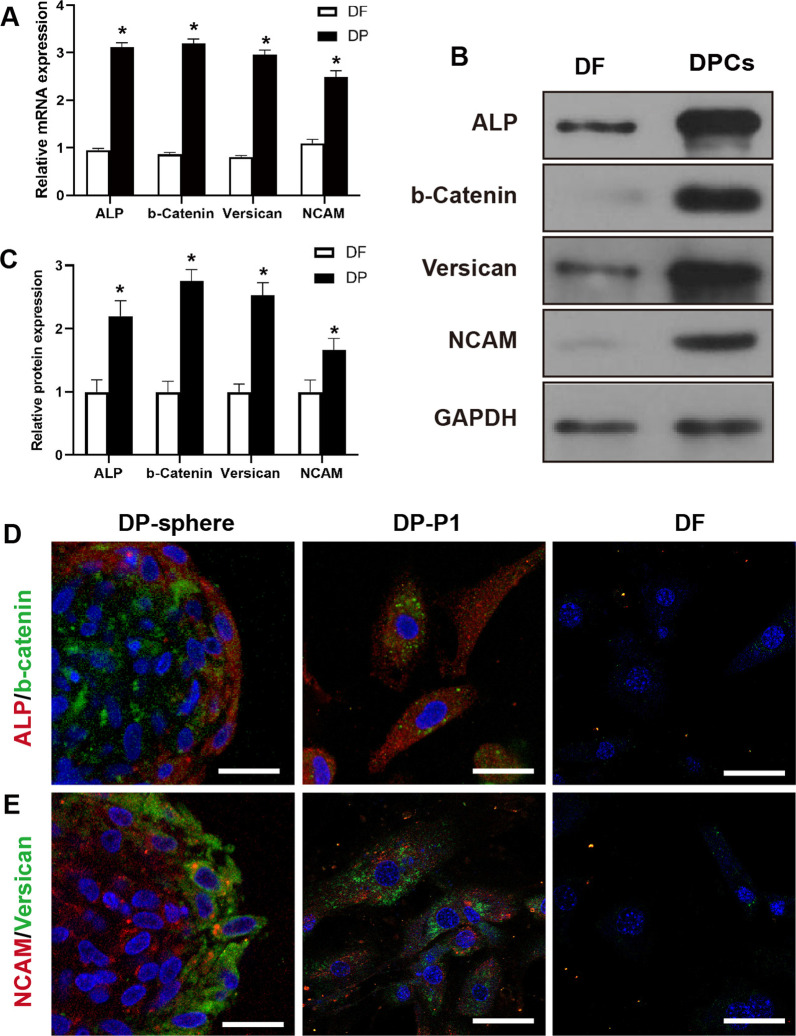
Characteristics of isolated DP cells in vitro. **A** Expression of genes in the DP cells associated with hair-inductivity, including ALP, b-Catenin, NCAM, and versican, (*p < 0.05). **B**, **C** Western blot analysis quantified the expression of these target proteins, which were in accord with the qPCR. DF cells were used as the control. Double-labeling immunofluorescence assay in DP-sphere, DP-P1 and fibroblasts with ALP and b-catenin (**D**), and NCAM and Versican (**E**), respectively. Scale bar: 10 μm

On the first day of culture, > 60% of DP-sphere explants had attached, with cells beginning to migrate away from the explants. After 3 days of culture, the DP-sphere explants were larger and flatter, with loss of initial structure. DP cells shown the differences morphology compared with DF (Fig. 3E). Immunostaining with the proliferative marker, Ki67, shown that the proliferative property of DP cells was not affected by two FDGS treatments (Fig. 3F). After the third passage, the DP cells were still proliferative (Fig. 3G). To confirm the efficiency of separation, we labeled freshly isolated dermal cells, NFC and DP cells with Sox2 antibodies, and sorted them by flow cytometry. Only 9.7% of dermal cells were Sox2^+^, and after the first FDGS treatment, up to 30.8% of NFC cells were Sox2^+^. After the second FDGS treatment, 85.7% of DP cells were positive (Fig. 3H, I).qPCR analysis was used to examine the expression of DP-specific genes, such as ALP, β-catenin, Versican and NCAM by qPCR analysis in DP cells and DF. As shown in Fig. 4A, ALP, β-catenin, Versican and NCAM were expressed in DP cells at significantly higher levels than in DF samples. The expression of ALP, β-catenin, Versican and NCAM marker proteins were confirmed by western blots, expression of all four proteins was increased in the DP cells group compared with controls in DF (Fig. 4B, C) (Full-length gels are presented in Additional file 1: Fig. S3). Consistent with the qPCR and WB analysis, significant differences in the expression of ALP, β-catenin, Versican and NCAM were also seen in immunofluorescence staining. All four proteins were expressed significantly higher in DP-spheres and first-passage DP cells, compared with DF control (Fig. 4D, E). It was concluded that purified DP-spheres could be successfully obtained from the NFC using differential centrifugation and purified by density gradient centrifugation. The resulting DP cells retained their adherent and proliferative capacity, and expressed characteristic HF induction markers.

### *Characteristics of isolated DP cells *in vivo

**Fig. 5:**
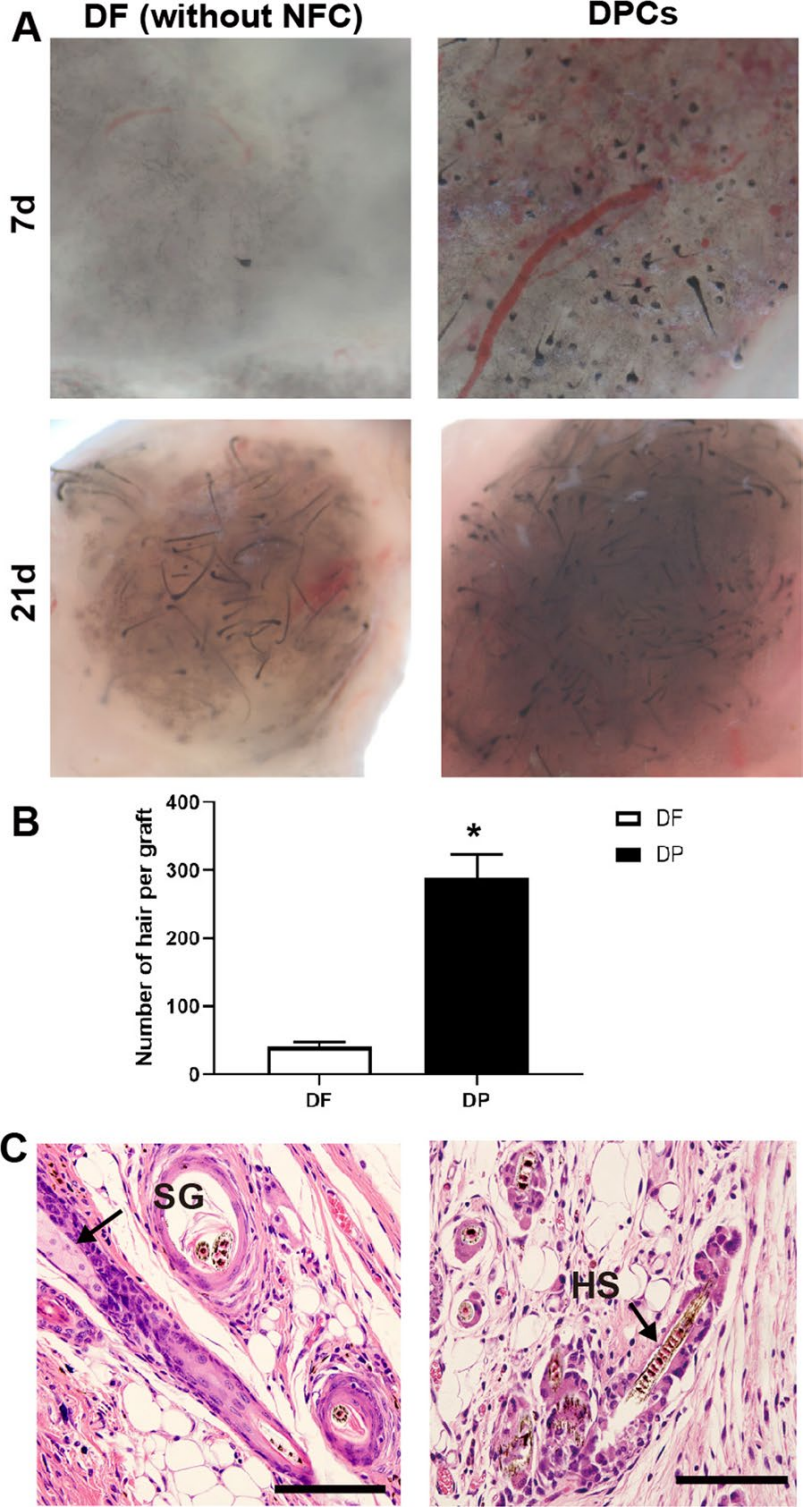
Characteristics of isolated DP cells in vivo. **A** The results of the chamber assay showed that DF cells (without an NFC) induced few HFs, while DP cells induced more HFs. **B** The number of hairs induced by DP cells was significantly higher than that induced by DF cells after FDGS treatment and without an NFC, (*p < 0.05). **C** H&E staining showed that the organization of these newly formed follicles resembled a mature follicle. Scale bar: 50 μm

To investigate whether isolated DP cells could generate HFs in vivo, the isolated DP cells after FDGS treatment were mixed with newborn mice epidermal cells and subcutaneously implanted into adult nude mice. After 1 weeks of implantation, newly formed HFs and regenerated hair shafts were observed within the hypodermis of the nude mice. However, when DF cells were implanted with epidermal cells in the absence of the NFC, only few hair shaft regeneration and large melanin granules were observed (Fig. 5A). After 3 weeks, the number of hairs induced by DP cells was significantly greater than DF (Fig. 5B). Histological sections of the skin at the transplantation site showed that these newly formed follicles resembled a mature HF (Fig. 5C).

## Discussion

In this study, we reported a simple and improved method named FDGS as a novel method to isolate and purify DP cells from neonatal mouse skin.

The hallmark of DFs was their ability to synthesize the extensive extracellular matrix, especially types I and III collagens, to fill the intercellular space in the dermis [25]. However, DP cells can synthesize a type IV collagen that resembles the basement membrane matrix [26, 27]. We used collagenase I to specifically digest the type I collagen in the mouse dermis to obtain single fibroblasts and NFCs, the latter of which retained their 3D structures.

Due to the differences between single fibroblasts and NFCs (such as cellularity, density, and weight), NFCs can be isolated and purified using different centrifugal force and density gradient centrifugations. According to the basic principles of centrifugation, repeated low-speed centrifugation primarily pelleted NFC. However, the resulting pellet was still contaminated with some fibroblasts. Density gradient centrifugation is a technique that separates cells according to their density. Ficoll PM400, a highly branched polymer formed by the copolymerisation of sucrose and epichlorohydrin, is the most commonly used density gradient medium [28–30]. NFCs can be purified by removing unwanted fibroblasts using the Ficoll gradient centrifugation. In addition, this centrifugation pattern is also applicable to the subsequent separation and purification of DP-spheres by applying appropriate centrifugal force. After separation and purification, the resulting DP-spheres showed favorable adherent and proliferative capacity for implantation, and expressed characteristic markers including ALP, β-catenin, versican, and NCAM.

The FDGS technique has several significant advantages over current methods. Primarily, FDGS can obtain a large number of DP cells from neonatal murine pelage follicles without the use of microdissection or FACS. FDGS was not limited by sample size, so it can separate neonatal murine pelage follicles, which compensated for difficulty of HF-DP cell isolation. Moreover, the FDGS method minimized the air exposure during DP cell isolation, which may reduce potential contamination. This therefore ensured cell-survival rate and reduced the loss of cellular characteristics. The Use of the FDGS technique may reduced the need to use transgenic animals or fluorochrome-conjugated antibodies, which means minimal training or preparation. However, FDGS is not considered to be superior to the flow cytometry technique. In the condition of sufficient transgenic mice and surface markers, flow cytometry technology is undoubtedly a more accurate research means. Secondary, many in vivo models have been developed to investigate hair regeneration, including the patch, chamber, and sandwich assays [31]. These methods were useful to study the developmental biology and tissue regeneration of hair. However, a remaining problem was how to obtain large numbers of DP cells. Using FDGS, 15–20 skin samples from mouse pups can be handled at one time, yielding approximately 1.08 ± 0.51 × 10^5^ DP cells per skin sample (Table 1). Furthermore, our results showed that the isolated DP cells maintained favorable hair inductive ability in vivo. Thirdly, we successfully separated NFCs from the dermis, as well as DP-spheres from an in vitro cultured NFC cell suspension, by modulating centrifugal forces and centrifugation procedures. Based on this evidence, the FDGS method could be used to isolate adult murine pelage or human scalp HF cells by adapting an appropriate centrifugal force. Subsequently, we successfully isolated human DP-spheres from the scalp follicle by using a modified FDGS method (Additional file 1: Fig. S2).

**Table 1:** Various cell preparation yields in FDGS isolation protocol

Source	Cell type	Yield	Unit(cells/per pup)
Dermis	Dermal fibroblasts	4.12 ± 0.62	10^7^
NFC	DP + other follicle cells	2.74 ± 0.33	10^6^
DP sphere	DP cells	1.08 ± 0.51	10^5^

In addition to their importance in regulating hair growth cycle and reconstruction, DP cells are also suitable for other research applications. Murine DP cells, which endogenously express the pluripotent transcription factors (TFs) Sox2, c-Myc, and Klf4 [32] can be reprogrammed into induced pluripotent stem (iPS) cells by either a single (Oct4) [33] or two TFs (Oct4 and Klf4) [34]. This suggested that DP cells represented a somatic cell source for iPSC generation that was easily obtained from skin.

## Conclusion

The optimized FDGS technique allows for efficient isolation of DP cells from neonatal mouse pelage follicles. This method not only provides a better source of DP cells, but also improves their research potential for tissue engineering.

## Supplementary Information

**Additional file 1**. Supplementary experimental procedures and figures.

## Data Availability

The data used to support the findings of this study are available from the corresponding author upon request.

## Abbreviations

FDGS: Ficoll density gradient sedimentation
HFs: Hair follicles
NFC: Naïve follicle components
DP: Dermal papilla
DF: Dermal fibroblast
