# Supplementary material for: An optimized force-triggered density gradient sedimentation method for isolation of pelage follicle dermal papilla cells from neonatal mouse skin
Source: Stem Cell Res Ther. 2023 May 24;14:140. doi: 10.1186/s13287-023-03343-2 (PMC10210473; doi:10.1186/s13287-023-03343-2)
Supplement: Supplementary file 1 — Additional file 1. Supplementary experimental procedures and figures. [file 13287_2023_3343_MOESM1_ESM.doc]

An optimised force-triggered density gradient sedimentation method for isolation of pelage follicle dermal papilla cells from neonatal mouse skin

Lijuan Du^a^, Yuyang Gan^a^, Bowen Zheng, Junfei Huang, Zhiqi Hu^+^ and Yong Miao^+^

Department of Plastic and Aesthetic Surgery, Nan Fang Hospital of Southern Medical University, 1838 North Guangzhou AV, Guangzhou, Guangdong, China;

**^a^** These two authors contributed equally to this work

***** Correspondence: [18688845651@163.com](mailto:18688845651@163.com) (Y.M); [doctorhzq@hotmail.com](mailto:doctorhzq@hotmail.com) (ZQ.H)

Supplemental experimental procedures

1. Cellular grafting for reconstructing HFs

The cellular grafting procedure for reconstituting HFs *in vivo* was performed[1]. In brief, mouse skins were derived from newborn C57BL/6 mice pups. Suspensions of 10^6^ freshly isolated DP and DF cells (without an NFC) were mixed with equal volumes of fresh epidermal cells in 50 μL DMEM. DF cells in the absence of NFC suspensions mixed with an equal volume of epidermal cells were used as a negative control.

Male athymic nude mice were age-(4 week old) and weight- (25–30 g) matched and randomly assigned to each experimental group (n=3/group/cage). Blinding was not performed. Mice were anesthetized with pentobarbital (50 mg/kg) through intraperitoneal injection. A 100 μL mixed cell suspension was injected subcutaneously into the dorsal skin with 29-gauge needles. The mice were kept under a warming lamp after transplant procedure until they recovered from anesthesia. HF formation of each transplant site was monitored every 24 h for 3 weeks. Animals that died within 24 h after the transplant procedure were removed from the study. Animal behavioral and physiological parameters were monitored daily and suffering animals (e.g., emaciation, infection, stereotyped behavior) were euthanized for humane reasons. The mice were killed to harvest tissues at D 7 and D 21 after transplantation. The number of HF formed in transplant site was measured.

2. Quantitative (q) RT-PCR analysis

Total RNA was extracted from DP cells using RNAiso Plus reagent (TaKaRa, Dalian, China). cDNA was synthesised from 2 μg total RNA with a SYBR PrimeScript RT-PCR Kit (TaKaRa). qPCR analysis was performed using a SYBR PrimeScript RT-PCR Kit on a Stratagene MX3005 P qPCR system (Agilent Technologies, Santa Clara, CA, USA) following the manufacturer’s protocol. The fold change of each target gene was normalized to GAPDH expression.

3. Western blotting analysis

For western blotting, cell lysates were obtained using RIPA lysis buffer (Gibco), and 30 μg aliquots of total protein from each sample was subjected to sodium dodecyl sulphate polyacrylamide gel electrophoresis and transferred to a polyvinylidene fluoride membrane. After blocking, the blotted membranes were incubated with the following primary antibodies at 4 °C overnight: ALP (1:10000), β-catenin (1:5000); Versican (1:5000); NCAM(1:5000) and GAPDH (1:1000) monoclonal antibody. The blots were incubated with corresponding secondary antibodies (1:5000). The immune complexes were assayed with an enhanced chemiluminescence kit (Invitrogen) and analyst/PC densitometry software (Bio-Rad Laboratories, Hercules, CA, USA).

4. Histological analysis

After mice were euthanized with CO2 chamber, the dermal skin specimens were fixed in 4% paraformaldehyde in PBS for 60 min, washed and embedded in paraffin. Paraffin-embedded skin tissues were cut in 5 μm sections with a microtome. Sections were stained with H&E using a standard protocol[2].

5. Immunofluorescence staining

Cultured cells and tissue sections were fixed in 4% paraformaldehyde (Gibco) for 30 min at 4°C. After washing three times with PBS (Gibco), the samples were permeabilized with 5% Triton X-100 (Sigma) for 10 min, followed by blocking in 5% Bovine serum albumin (Gibco) in PBS for 30 min. Primary antibodies used were as follows: rat anti-Sox2 (1:100, Thermo Fisher, Waltham, MA, USA), rabbit anti-K14 (1:1000, Abcam), ALP (1:100, Abcam), b-catenin (1:200, Abcam), NCAM (1:250, Abcam) and versican (1:200, Abcam). After primary antibody incubation overnight at 4°C, cells were washed twice with PBS before incubation with secondary antibodies for 60 min at room temperature. Secondary antibodies used were as follows: goat anti-rat Alexa-Fluor-488 and 594, or goat anti-rabbit Alexa-Fluor-488 and 594 (1:250, Abcam). All samples were counterstained with DAPI ReadyProbes^TM^ (2 drop per mL, Thermo Fihser) for 10 min. Images were recorded using a fluorescent microscope (IX71 FL, Olympus, Japan) and confocal laser scanning microscope (Olympus FLUOVIEW FV10i). Z-stacks were acquired at 100 Hz with an optimal stack distance and 1024 × 1024 dpi resolution. Z-stack projections were generated using the Olympus FV10-ASW software package.

1. Flow cytometric analysis

Single cell suspensions prepared from freshly isolated dermal cells, NFC, and DP spheres were treated with 0.25% trypsin, then strained through a 40-μm cell strainer. Dermal cells, NFC and DP cells were fixed with 4% paraformaldehyde for 15 min and permeabilized with 0.2% Tween 20 (Abcam) for 15 min, followed by blocking in 5% Bovine serum albumin (Gibco) in PBS for 30 min. Cell suspensions were incubated with rabbit anti-Sox2 antibody (1/200, Abcam) at 4°C for 30 min, followed by treatment for 15 min at 4°C with goat anti-rabbit Alexa-Fluor-488 (1:200; Abcam). After staining, cells were sorted by a FACS Aria Flow Cytometer (BD Biocsiences, San Jose, CA, USA).

**References:**

1. Lin B, Miao Y, Wang J, Fan Z, Du L, Su Y, et al. Surface Tension Guided Hanging-Drop: Producing Controllable 3D Spheroid of High-Passaged Human Dermal Papilla Cells and Forming Inductive Microtissues for Hair-Follicle Regeneration. Acs Appl Mater Inter. 2016;8(9):5906-16
2. Gan Y, Wang H, Du L, Fan Z, Sun P, Li K, et al. Ficoll density gradient sedimentation isolation of pelage hair follicle mesenchymal stem cells from adult mouse back skin: a novel method for hair follicle mesenchymal stem cells isolation. Stem Cell Res Ther. 2022;13(1):372.

Supplemental figure

**
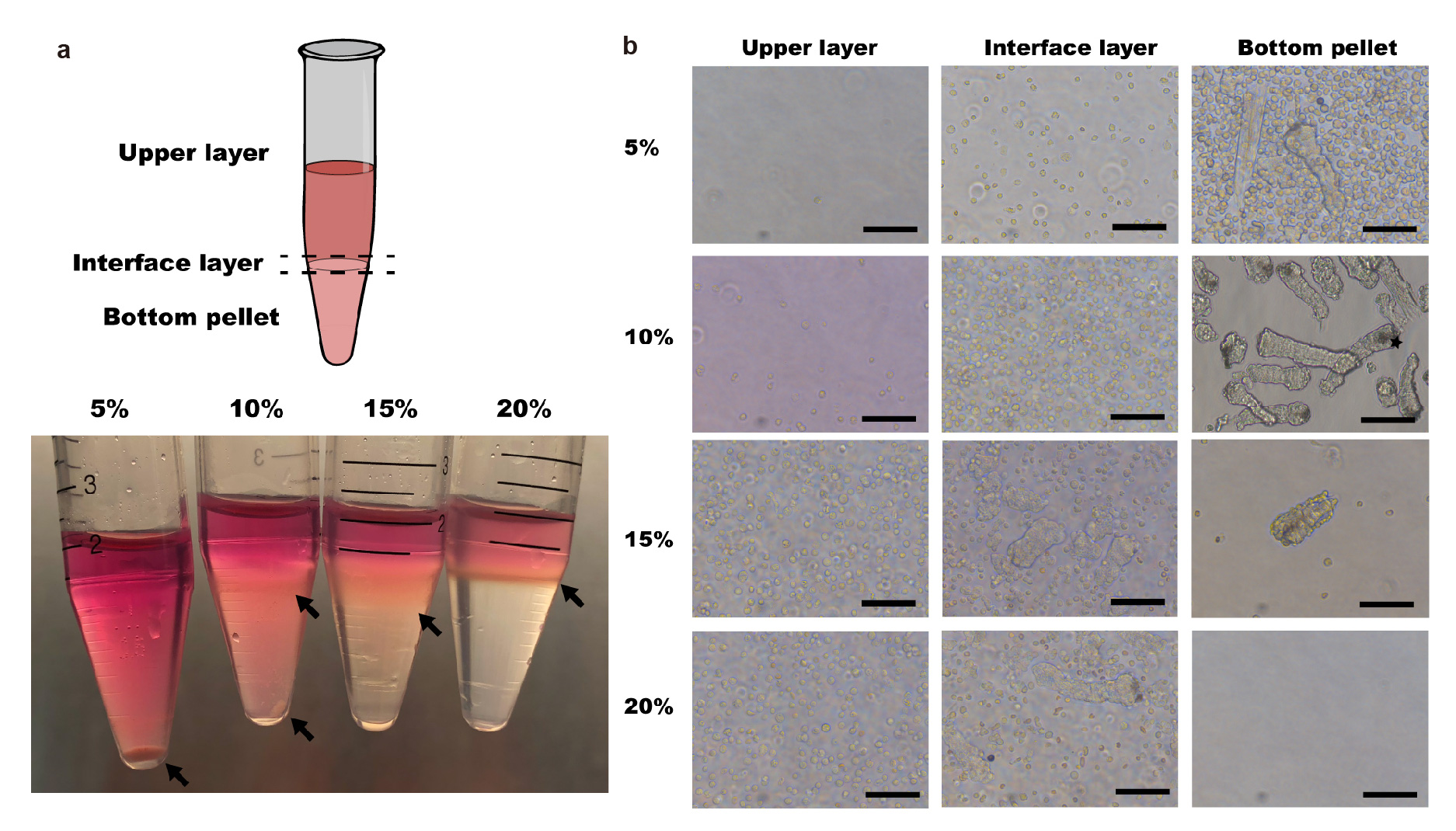
**

**Figure S1.** Ficoll concentration optimisation for NFC separation. (a) Photographs of different bilayer discontinuous Ficoll density gradients (5%, 10%, 15%, and 20%) overlayed with a mixed dermal cell suspension after an initial FDGS treatment. (b) Representative images of NFC before and after FDGS1st treatment. The collection of NFC micro-tissue was greatest with a 10% Ficoll gradient, and then decreased in a dose-dependent manner. Asterisks denote DP-spheres. Scale bars: 50 μm.

**
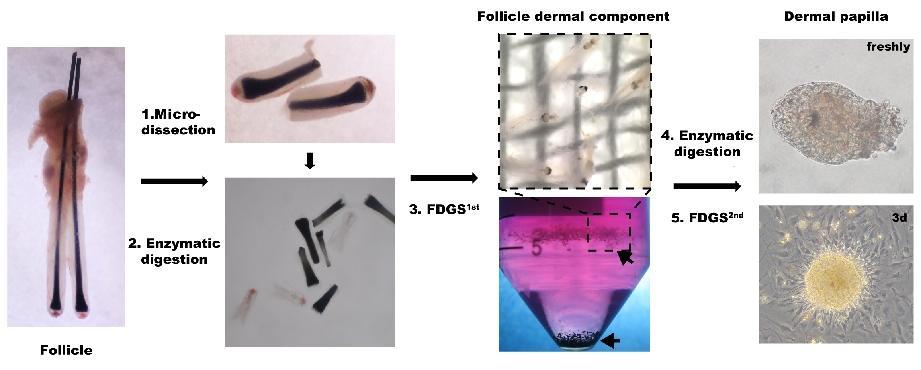
**

**Figure S2.** The harvest of dermal papilla from human scalp hair follicles by modified FDGS treatment. Human scalp hair follicles were obtained by follicular unit extraction. The dermal component was obtained using either enzymatic digestion followed by microdissection. DP-spheres were obtained with a modified FDGS protocol.


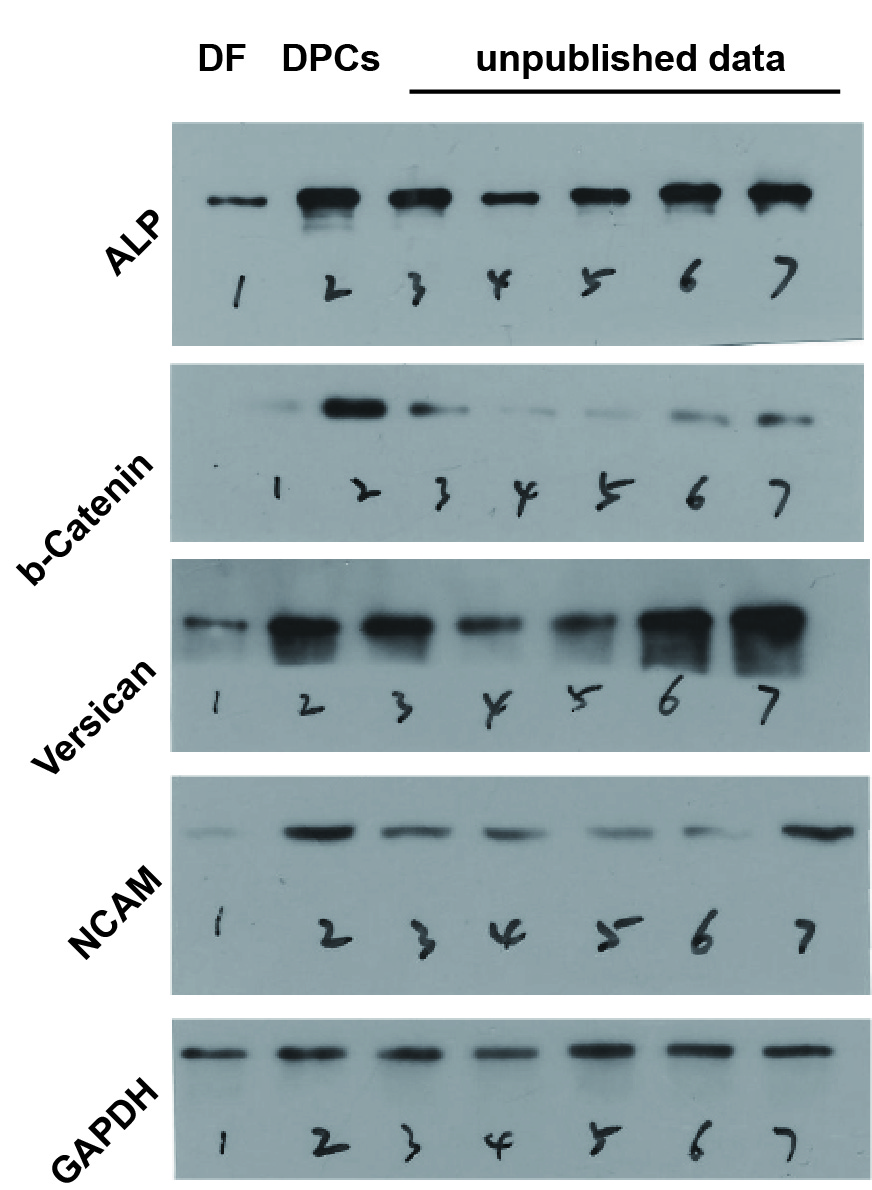


**Figure 3.** Full-length gels of western blots
